# Heat Resistant Characteristics of Major Royal Jelly Protein 1 (MRJP1) Oligomer

**DOI:** 10.1371/journal.pone.0119169

**Published:** 2015-05-28

**Authors:** Takanori Moriyama, Aimi Ito, Sumire Omote, Yuri Miura, Hiroki Tsumoto

**Affiliations:** 1 Faculty of Health Sciences, Hokkaido University, Kita-ku, Sapporo, Japan; 2 Division of Clinical Laboratories, Toranomon Hospital, Kawasaki, Japan; 3 Graduate School of Health Sciences, Hokkaido University, Kita-ku, Sapporo, Japan; 4 Research Team for Mechanism of Aging, Tokyo Metropolitan Institute of Gerontology, Itabashi-ku, Tokyo, Japan; Nanyang Technological University, SINGAPORE

## Abstract

Soluble royal jelly protein is a candidate factor responsible for mammiferous cell proliferation. Major royal jelly protein 1 (MRJP1), which consists of oligomeric and monomeric forms, is an abundant proliferative protein in royal jelly. We previously reported that MRJP1 oligomer has biochemical heat resistance. Therefore, in the present study, we investigated the effects of several heat treatments (56, 65 and 96°C) on the proliferative activity of MRJP1 oligomer. Heat resistance studies showed that the oligomer molecular forms were slightly maintained until 56℃, but the molecular forms were converted to macromolecular heat-aggregated MRJP1 oligomer at 65℃ and 96℃. But, the growth activity of MRJP1 oligomer treated with 96°C was slightly attenuated when compared to unheated MRJP1 oligomer. On the other hand, the cell proliferation activity was preserved until 96℃ by the cell culture analysis of Jurkat cells. In contrast, those of IEC-6 cells were not preserved even at 56°C. The present observations suggest that the bioactive heat-resistance properties were different by the origin of the cells. The cell proliferation analysis showed that MRJP1 oligomer, but not MRJP2 and MRJP3, significantly increased cell numbers, suggesting that MRJP1 oligomer is the predominant proliferation factor for mammiferous cells.

## Introduction

Royal jelly is secreted from the hypopharyngeal and mandibular glands of nurse honeybees [1, 2], and it plays a specific and important role in queen honeybee development [3]. The queen honeybee is fed royal jelly throughout the larval period, while nurse honeybees are fed royal jelly for only 3 days. Royal jelly contains various components: 60–70% moisture content; 12–15% crude proteins; 10–16% total sugar; and 3–6% lipids, vitamins, salts and free amino acids [3–5].

One of the physiological functions of royal jelly in mammals is cell proliferation activity [6, 7]. The candidate factor for increasing cell proliferation is a soluble royal jelly protein, and major royal jelly protein 1 (MRJP1) is the predominant factor exerting mammiferous cell growth [8–12]. In these previous reports, we demonstrated dose-dependent proliferation activity in cultures of a human cell line (Jurkat cells) with purified MRJP1 oligomer [8]. Among soluble royal jelly proteins, more than 80% belong to the MRJP family [3, 13, 14], which includes nine members, MRJP 1–9 [15, 16]. MRJP1 is the most abundant protein within this family, comprising MRJP1 oligomer, a 280-420-kDa oligomeric hetero complex with Apisimin [8, 17], or MRJP1 monomer, a 55-kDa monomeric form also known as apalbumin1 or royalactin [9, 18]. Interestingly, Kamakura reported that MRJP1 monomer (royalactin) is the crucial factor in queen honeybee development [19].

Previous reports have shown that the contents and physiological functions of royal jelly are altered in accordance with storage conditions (-20°C to 50°C). Proteins and simple sugars in royal jelly change significantly during storage at room temperature, but not at -20°C [4]. With regard to soluble proteins in RJ, a 57-kDa protein known as MRJP1 monomer or royalactin is gradually degraded under various temperature conditions from 4°C to 50°C [19, 20]. Moreover, the stability of MRJP1, MRJP2 (apalbumin 2) and MRJP3 (apalbumin 3) is sensitive to storage temperature: -20°C, 4°C and room temperature, respectively [21]. However, it has remained unclear whether high-temperature conditions affect the biochemical or functional characteristics of royal jelly proteins.

Previously, we demonstrated that the oligomeric conformation of MRJP1 was preserved after heat treatment at 56°C [8]. This finding has led to the hypothesis that the physiological function of MRJP1 oligomer is preserved after heat treatment. In the present study, to examine whether heat treatment affects the bioactivity of MRJP1 oligomer, we investigated the cell growth activity of MRJP1 oligomer heated at 56°C, 65°C or 96°C, which were selected based on the work of Watanabe et al. [12] and Shen [28].

## Materials and Methods

### Research materials

Fresh royal jelly was provided by Japan Royal Jelly Co., Ltd. (Tokyo, Japan). Samples were preserved at -80°C until analysis.

### Extraction of soluble royal jelly proteins

Extraction of soluble royal jelly proteins was performed in accordance with our previous report [22]. Briefly, royal jelly was dissolved in deionized water and mixed using a vortex mixer. Royal jelly solution was ultracentrifuged at 113,400 × g for 1 hour at 4°C using an Optima^™^ L-80XP Ultracentrifuge (Beckman Coulter, Tokyo, Japan). The three supernatant layers were then collected as the upper, middle and lower layers, and the layered protein fractions were designated as upper-, middle- or lower-layered soluble royal jelly proteins (LSRJPs). These fractionated proteins were stored at -80°C until further analysis.

### Measurement of total protein

Total protein concentration in samples was quantified using a Micro BCA protein Assay Kit (Pierce, Rockford, IL). Human serum albumin solution (Wako, Osaka, Japan) was used as a protein standard.

### High-pressure liquid chromatography techniques

**Fig 1:**
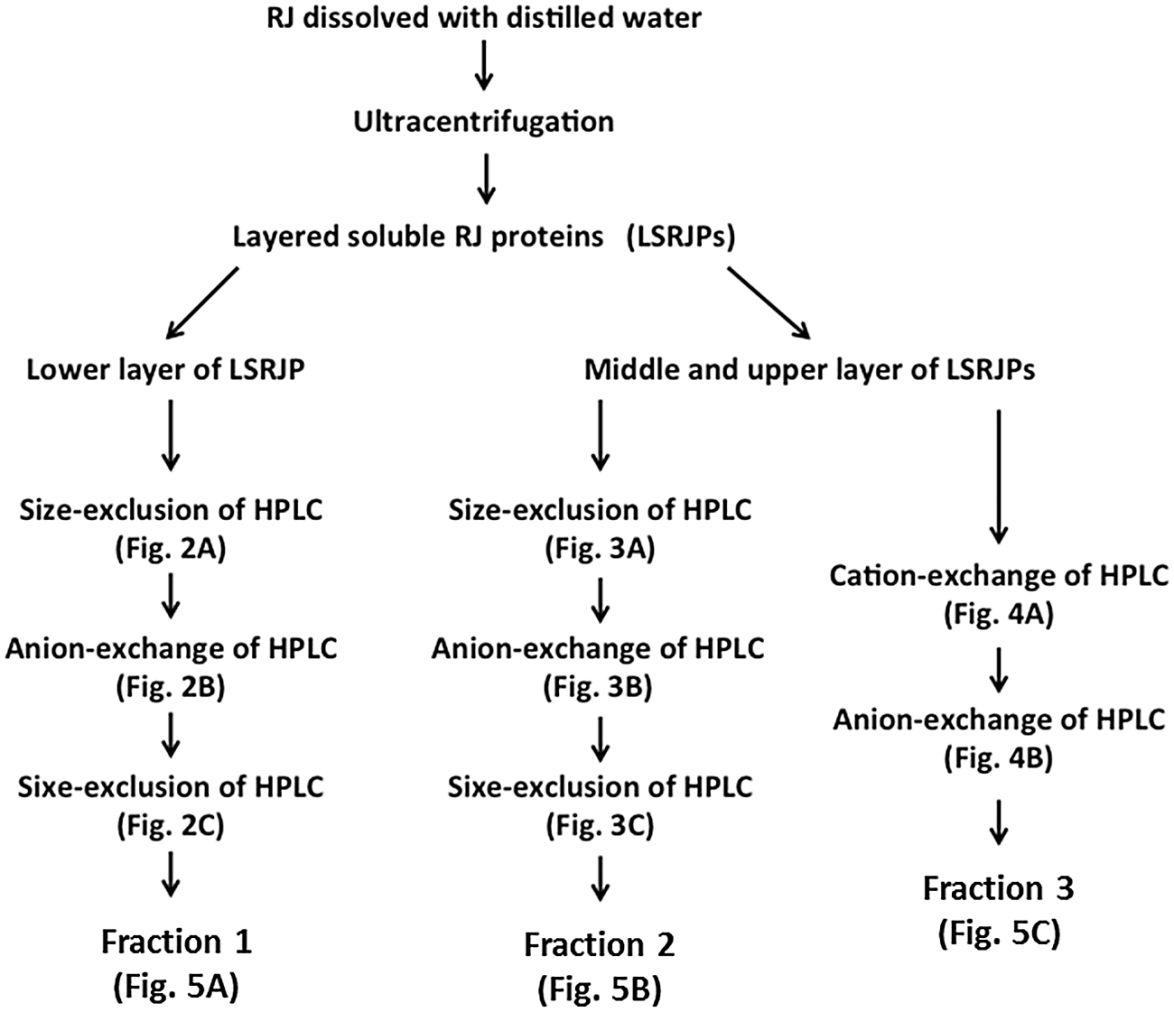
Separation scheme of each MRJP from LSRJPs.

MRJP1 oligomer and MRJP2 were separated using size-exclusion and anion-exchange HPLC. MRJP3 was isolated using cation-exchange and anion-exchange HPLC. These chromatographic steps are summarized in Fig 1.

Size-exclusion HPLC was performed using Sephacryl S-200 (16×600 mm; GE Healthcare, Buckinghamshire, UK). Elusion buffer was phosphate buffer saline (20 mM Na_2_HPO_4_, 2 mM NaH_2_PO_4_·2H_2_O and 150 mM NaCl, pH 7.4). For the Sephacryl S-200 column, the sample injection volume was 2 ml and flow rate was 0.4 ml/min.

Anion-exchange HPLC was performed on a Mono Q column (4.6×100 mm, GE Healthcare). Sample desalting and buffer exchange was carried out by dialysis with 20 mM Tris-HCl (pH 8.0). Condensation of protein was carried out using an Amicon Ultra-4 (Millipore, Billerica, MA). The sample injection volume was 10 ml. Binding buffer was 20 mM Tris- HCl (pH 8.0) and elution buffer was 20 mM Tris-HCl, 1.0 M NaCl (pH 8.0). Protein elusion from the Mono Q column was performed with a liner NaCl gradient from 0 to 0.5 M NaCl. The flow rate was 1.0 ml/min and the fraction volume was 2.0 ml.

Cation-exchange HPLC was performed on a Mono S column (4.6×100 mm; GE Healthcare). The sample was dialyzed against deionized water and diluted ten-fold with binding buffer. Binding buffer was phosphate buffer (20 mM Na_2_HPO_4_, 2 mM NaH_2_PO_4_·2H_2_O, pH 7.4) and the sample infection volume was 10 ml. Elution buffer was 20 mM Na_2_HPO_4_, 2 mM NaH_2_PO_4_·2H_2_O and 1.0 M NaCl (pH 7.4). Protein elusion from the Mono S column was performed with a linear NaCl gradient from 0 to 0.5 M NaCl. The flow rate was 2.0 ml/min and fraction volume was 1.0 ml. Purified protein was dialyzed against distilled water and lyophilized.

Heat treatment samples of MRJP1 oligomer were subjected to size-exclusion HPLC with a Superose 12 column (10×300 mm, GE Healthcare). Elusion buffer was PBS, as described above. The flow rate was 0.5 ml/min and the fraction volume was 0.8 ml. All HPLC techniques were carried out using an Akta Explorer System (GE Healthcare). Protein elution profiles were monitored at 280 nm.

### Two-dimensional electrophoresis (2-DE)

One and/or twenty microgram of protein was dissolved with protein solubilizer 1 (Invitrogen), carrier ampholytes 3–10 (Invitrogen, Tokyo, Japan) and 5 mM dithiothreitol (DTT). Protein solution was added to an IPG ZOOM strip gel (pH 3–10; Invitrogen) followed by incubation overnight at room temperature. First-dimension isoelectric focusing (IEF) was run under gradient voltage conditions (175 V constant for 20 min, gradient from 175 to 2000 V for 45 min, and 2000 V constant for 30 min). The IPG strip gel used for IEF was reduced with 50 mM DTT in lithium dodecylsulfate (LDS) sample buffer and was alkylated with 125 mM iodoacetamide in LDS sample buffer. The incubated strip gel was applied to a precast NuPAGE 4–12% bis-Tris polyacrylamide gel (Invitrogen) as a second-dimension sodium dodecyl sulfate polyacrylamide gel electrophoresis (SDS-PAGE). NuPAGE MES SDS running buffer (Invitrogen) was used as running buffer, and electrophoresis was performed at 200 V. CandyCane glycoprotein molecular weight standards (Invitrogen) were used as a molecular weight marker. One microgram of protein applied 2-DE gel was subjected to SYPRO Ruby protein gel stain (Invitrogen), 20 microgram of protein applied 2-DE gel was subjected to Coomassie Brilliant Blue (CBB) R-250 protein gel stain. The products isolated using HPLC techniques were evaluated by the former electrophoretic pattern. Those proteins identification were performed using the latter gel. Briefly, the spots visualized by CBB R-250 stain were subjected to the following in gel digestion. The protein detection limit of SYPRO Ruby is 0.25 to 1 ng per spot. In accordance with the manufacturer’s instructions, gels were fixed, stained and washed. Protein signals were detected using the VersaDoc Imaging System 5000 (Bio-Rad, Hercules, CA).

### Mass spectrometry and protein identification

In-gel digestion on the selected gel spots was performed using XL-TrypKit (APRO Science Inc., Tokushima, Japan) according to the manufacturer’s instructions. Matrix-assisted laser desorption ionization time of flight/ time of flight of mass spectrophotometer (MALDI-TOF/TOF MS) analyses were carried out using MALDI-TOF/TOF 5800 (AB sciex), as described previously [23]. MS spectra were acquired from *m/z* 800 to 4000, and each spectrum was obtained by accumulating 800 laser shots. Bradykinin fragment 2–9 (*m/z* 904.47), angiotensin I (*m/z* 1296.69), Glu^1^-fibrinopeptide B (*m/z* 1570.68), ACTH fragment 1–17 (*m/z* 2093.09), ACTH fragment 18–39 (*m/z* 2465.20), and ACTH fragment 7–38 (*m/z* 3657.93) were used for external calibration. MS/MS spectra were automatically acquired from the intensive precursor ions (S/N > 50). Protein identification by MS/MS ion search (MIS) was performed using ProteinPilot software (ver.4.5; AB sciex). Search parameters were as follows: database, uniprot_sprot_can+iso_20100622; species, none; Cys alkylation, iodoacetamide; digestion, trypsin; and special factors, Gel-based-ID, max missed cleavage, 1. Protein identification was considered to be correct based on the following selection criteria: protein having at least 2 peptides with an ion score above 95% confidence; and protein with protein score (ProtScore) > 1.3 (unused, p < 0.05, 95% confidence).

### Heat treatment of MRJP 1 oligomer and sterilization

Lyophilized MRJP 1 oligomer was gently dissolved in phosphate buffer saline and sterilized using Ultrafree-MC Centrifugal Filter Devices (Millipore). Two hundred microliters of 5 μg/μl MRJP1 oligomer solution was divided into 1.5-ml polypropylene sample tubes (Product No. A151; ASSIST, Tokyo, Japan). Heating was carried out for 30 min using CHILL HEAT CHT-1000 (Asahi Glass Co., Ltd., Tokyo, Japan). Heating temperatures were 56°C, 65°C or 96°C, which are able to denature most non-covalent protein interactions, pasteurize liquid foods and disrupt protein structures, respectively. After heat treatment, samples were immediately subjected to electrophoretic analysis, Superose 12 HPLC analysis, and cell proliferation assay.

### Blue native-PAGE

Blue native-PAGE was performed using the Native PAGE Novex bis-Tris Gel System (Invitrogen). Five micrograms of protein analytes were mixed with NativePAGE sample buffer (Invitrogen) and NativePAGE 5% G-250 sample additive (Invitrogen). Mixtures were incubated for 30 min on ice. After incubation, analytes were applied to NativePAGE Novex 4–16% bis-Tris gel (Invitrogen), and electrophoresis was performed at 150 V. Preparation of running buffer and other conditions on Blue Native-PAGE were carried out in accordance with the manufacturer’s instructions. NativeMark unstained protein standard (Invitrogen) was used as a molecular weight marker for Blue Native-PAGE. Gels were stained with CBB R-250.

### SDS-PAGE

Five micrograms of protein analytes were mixed with NuPAGE LDS sample buffer (Invitrogen) containing 50 mM DTT, followed by boiling at 100°C for 5 min. Sample mixtures were applied to precast NuPAGE 4–12% bis-Tris polyacrylamide gels (Invitrogen). NuPAGE MES SDS running buffer (Invitrogen) was used as running buffer, and electrophoresis was performed at 200 V. Precision Plus Protein Dual Color Standards (Bio-Rad) were used as molecular weight markers. Gels were stained with CBB R-250.

### Cell culture and cell proliferation assay

MRJP1 proliferation assay was performed using Jurkat cells (human lymphoid cell line) [8] and IEC-6 cells (rat small intestine epithelial cell line). Briefly, Jurkat cells were cultured in RPMI-1640 medium (Sigma Aldrich, Tokyo, Japan) containing 5% fetal bovine serum (Invitrogen), 100 U/ml penicillin and 1100 mg/ml streptomycin (Invitrogen) at 37°C under a 5% CO_2_ atmosphere. Culture medium was exchanged with DME/F-12 medium (Sigma Aldrich) containing antibiotics, and cells were pre-cultured for 48 h prior to proliferation assay. Subsequently, cells were seeded into wells at 2×10^4^ cells/100 μL on 96-well plates (Nunc, Tokyo, Japan), and were cultured with 100 μl of sterilized MRJP 1 oligomer, heat-treated MRJP1 oligomer, MRJP2 or MRJP3. As a negative control, the same volume of phosphate buffer saline was added to the cell suspension. After incubation for 24 or 48 hours, cell proliferation activity was estimated by Alamar Blue assay (Invitrogen), in accordance with the manufacturer’s instructions.

IEC-6 cells were cultured in D-MEM (Wako, Tokyo, Japan) containing 10% fetal bovine serum (MP Biomedical), 1% Penicillin—Streptomycin Solution (Sigma Aldrich) at 37°C under a 5% CO_2_ atmosphere. Culture medium was exchanged with D-MEM (Wako) containing 1% Penicillin—Streptomycin solution, and cells were pre-cultured for 48 h prior to proliferation assay. Subsequently, cells were seeded into wells at 5×10^3^ cells/100 μL on 96-well plates (Corning, Tokyo, Japan), and were cultured with 100 μl of sterilized MRJP 1 oligomer, heat-treated MRJP1 oligomer, MRJP2 or MRJP3. As a negative control, the same volume of phosphate buffer saline was added to the cell suspension. After incubation for 24 hours, cell proliferation activity was estimated by water-solubule tetrazolium salt-8 (WST-8) assay (KISHIDA, Osaka, Japan), in accordance with the manufacturer’s instructions. Phase-contrast images of cells were observed by Leica DM IL LED phase-contrast microscopy (Leica, Tokyo, Japan).

### Statistical analysis

Values are given as means ± SD based on quintuplicate assays. Statistical analysis was performed by one-factor ANOVA. The level of significance was set at p<0.01 or p<0.05.

## Results

### Isolation of MRJP1 oligomer, MRJP2 and MRJP3 by HPLC techniques

MRJP1 oligomer, MRJP2 and MRJP3 were isolated from layered soluble royal jelly proteins (LSRJPs) by a combination of size-exclusion HPLC, anion-exchange HPLC or cation-exchange HPLC (Fig 1). Preparation of upper, middle and lower LSRJPs was performed as described previously [22].

**Fig 2:**
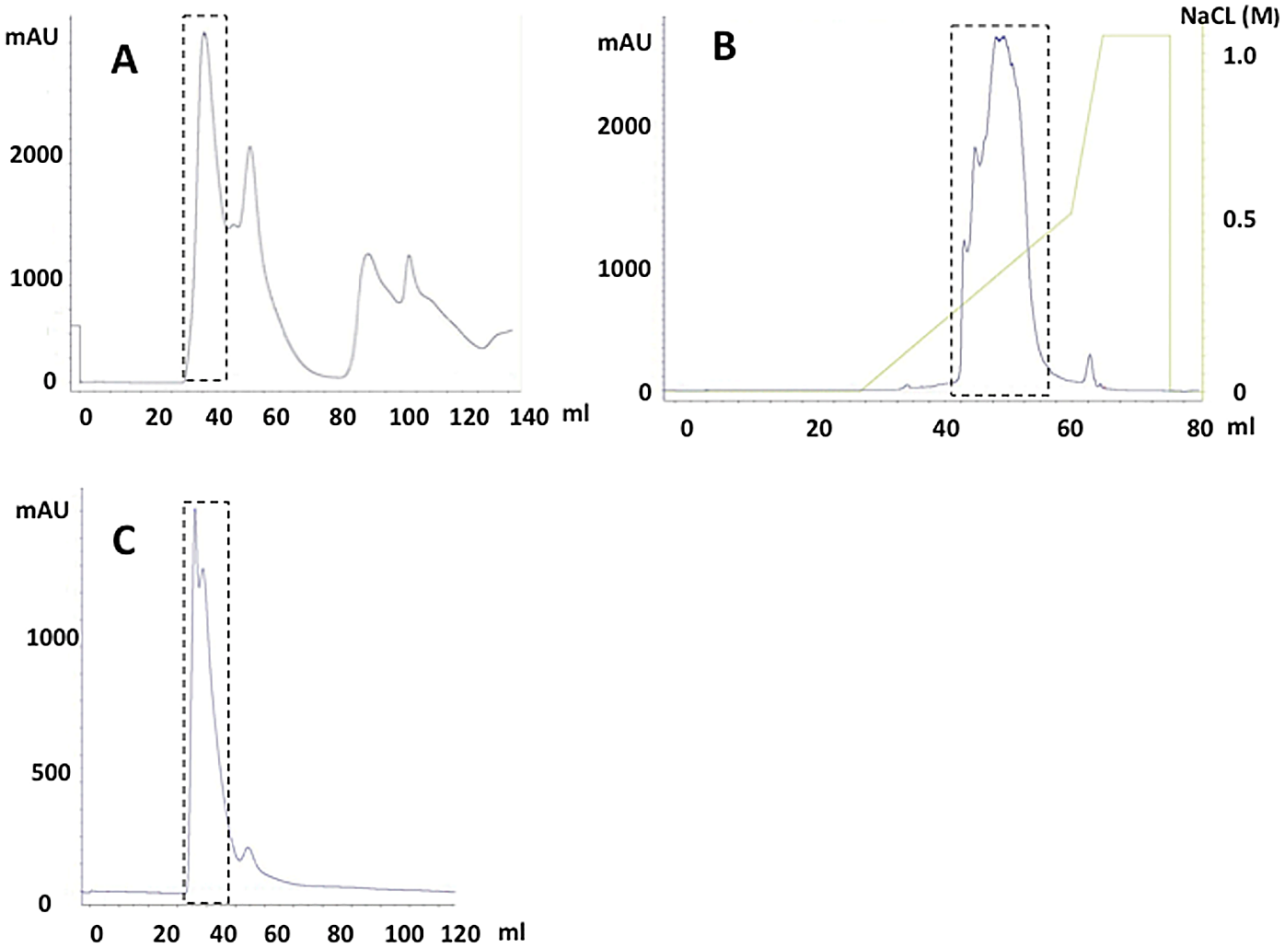
HPLC chromatographs of MRJP1 oligomer isolation. (A) The first isolation of MRJP1 oligomer from lower LSRJPs with Sephacryl S-200 size-exclusion HPLC. MRJP1 oligomer is eluted as a large peak at elution volume of 35 to 50 ml. (B) The second isolation of MRJP1 oligomer with Mono Q anion-exchange HPLC. MRJP1 oligomer is eluted at 0.2 to 0.4 M NaCl gradient. (C) The third isolation of MRJP1 oligomer with Sephacryl S-200 size-exclusion HPLC. The purified MRJP1 oligomer is eluted at elution time of 35 to 50 ml. The dashed line indicated those recovering fractions on each step.

MRJP1 oligomer was separated from lower LSRJPs in three isolation steps (Fig 2). Consistent with previous reports, MRJP1 oligomer was eluted as a 280-kDa peak on Sephacryl S-200 size-exclusion HPLC (Fig 2A). To eliminate other proteins in the MRJP1 oligomer fraction, Mono Q anion-exchange HPLC was carried out as a second isolation step. On Mono Q HPLC, MRJP1 oligomer was eluted using an NaCl gradient from 0.25 M to 0.40 M (Fig 2B). As a final purification, Sephacryl S-200 HPLC was repeated (Fig 2C). The large peak at 280 kDa on the third isolation with a Sephacryl S-200 column was collected and preserved as intact separated MRJP1 oligomer.

**Fig 3:**
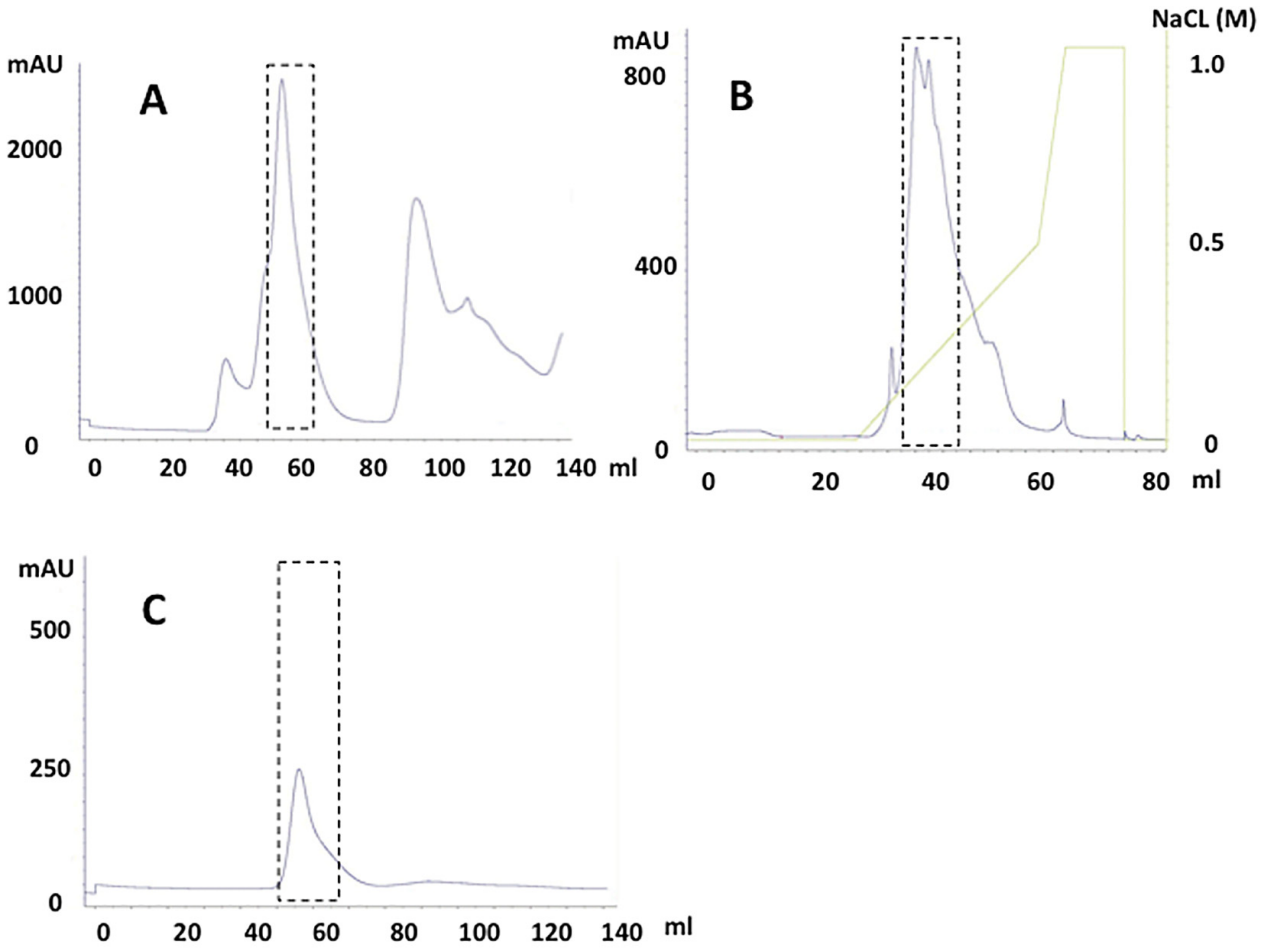
HPLC chromatographs of MRJP2 isolation. (A) The first isolation of MRJP2 from middle or upper LSRJPs with Sephacryl S-200 size-exclusion HPLC. MRJP2 is eluted at elution volume of 55 to 70 ml. (B) The second isolation of MRJP2 with Mono Q anion-exchange HPLC. MRJP2 is eluted at 0.15 to 0.25 M NaCl gradient. (C) The third isolation of MRJP2 with Sephacryl S-200 size-exclusion HPLC. The purified MRJP2 is eluted as a single peak at elution volume of 50 to 70 ml. The dashed line indicated those recovering fractions on each step.

MRJP2 was separated from the middle or upper LSRJPs in three isolation steps (Fig 3). Middle or upper LSRJPs were subjected to a first-step of Sephacryl S-200 size-exclusion HPLC. MRJP2 was eluted as a peak at approximately 70 kDa (Fig 3A). The MRJP2 fraction was subsequently subjected to a second step of Mono Q anion-exchange HPLC. MRJP2 was eluted using an NaCl gradient from 0.15 M to 0.25 M on Mono Q HPLC (Fig 3B). Finally, separated MRJP2 was further purified with a third step of Sephacryl S-200 size-exclusion HPLC (Fig 3C).

**Fig 4:**
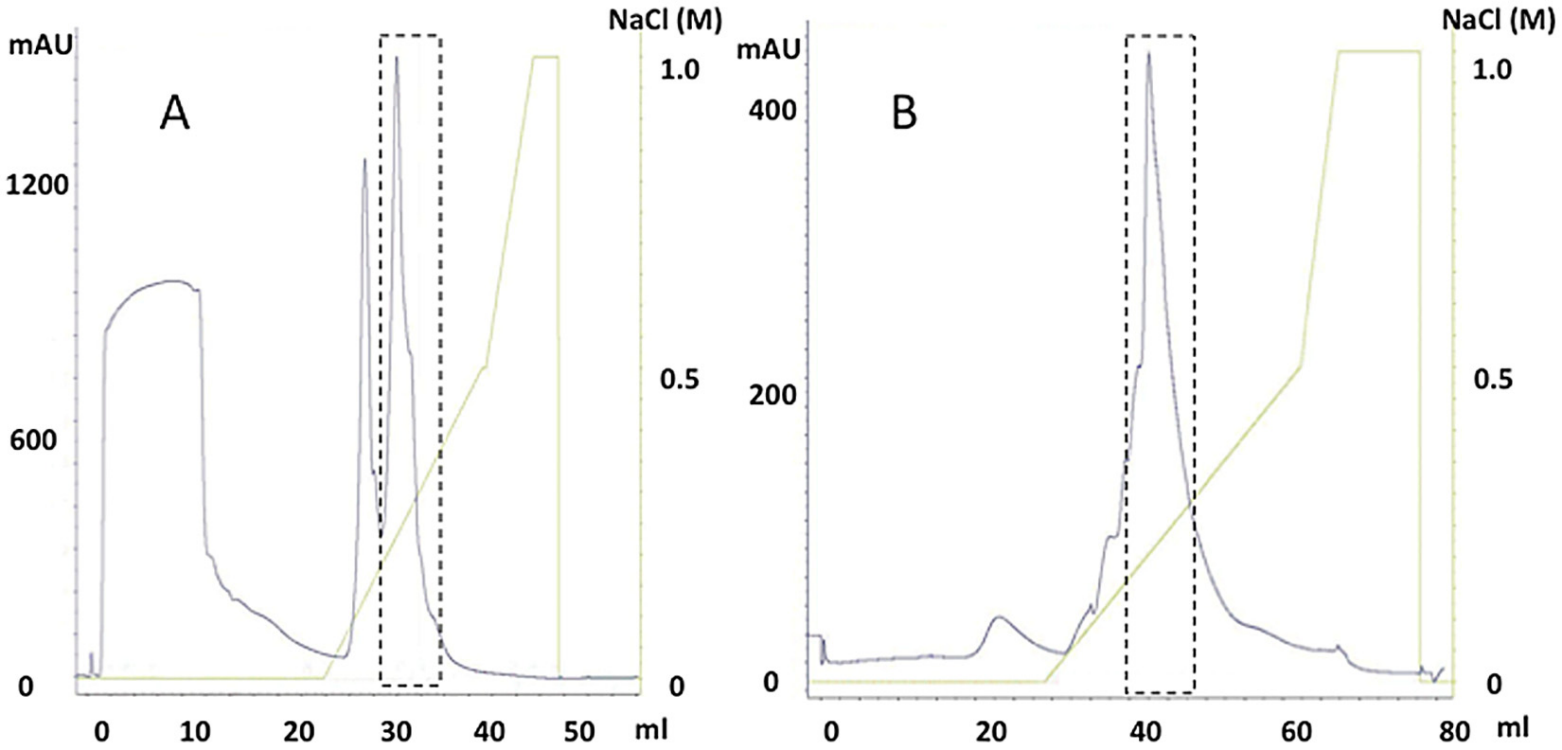
HPLC chromatographs of MRJP3 isolation. (A) The first isolation of MRJP3 with Mono S cation-exchange HPLC. MRJP3 is eluted at 0.20 to 0.30 M NaCl gradient. (B) The second isolation of MRJP3 with Mono Q anion-exchange HPLC. MRJP3 is eluted at 0.15 to 0.30 M NaCl gradient. A dashed square in each chromatograph indicates a elution peak of target protein. The dashed line indicated those recovering fractions on each step.

MRJP3 was separated from the middle or upper LSRJPs with two steps of ion-exchange HPLC (Fig 4), as size-exclusion HPLC is unsuitable for isolation of MRJP3 due to its molecular weight heterogeneity (60–70 kDa) [3] [24]. Upper or middle LSRJPs were subjected to a first-step of Mono S cation-exchange HPLC (Fig 4A). MRJP3 was eluted using an NaCl gradient from 0.20 to 0.30 M on Mono S HPLC. Subsequently, the MRJP3 fraction on Mono-S HPLC was subjected to a second-step of Mono Q anion-exchange HPLC (Fig 4B). MRJP3 was eluted using an NaCl gradient from 0.15 to 0.30 M on Mono Q HPLC.

### Quality assessment and Identification of separated MRJP1 oligomer, MRJP2 and MRJP3 by 2-DE

**Fig 5:**
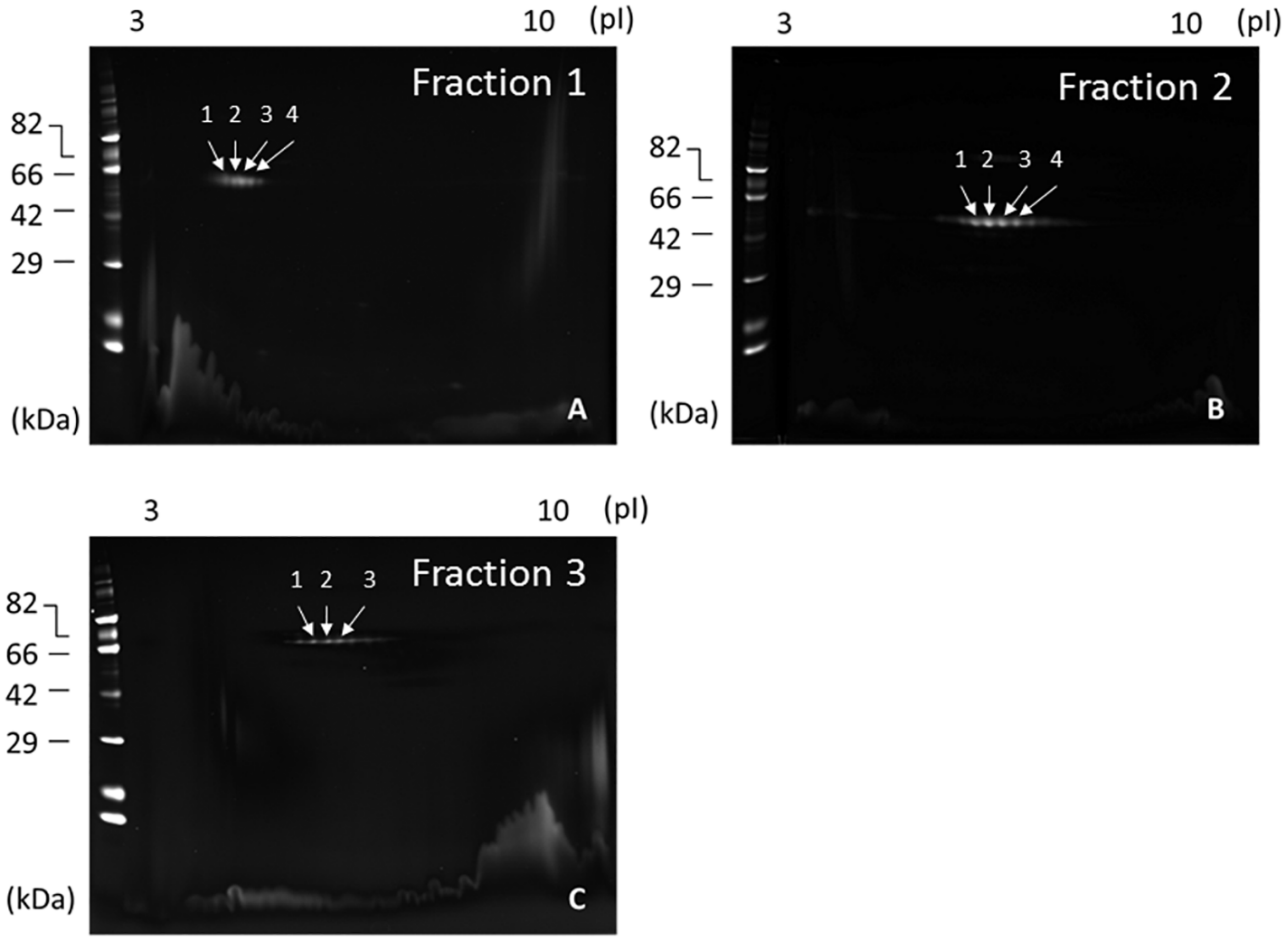
2-DE analysis to verify a quality of separated MRJP1 oligomer, MRJP2, and MRJP3. One micrograms of isolated MRJP1 oligomer (A), MRJP2 (B), and MRJP3 (C) were subjected to 2-DE with SYPRO Ruby staining to check an extra-protein contamination. These results indicate that there is no extra-protein contamination in separated each MRJP. The number of arrows is equivalent to the spot number shown in Table 1.

As our protein separation system was based on HPLC techniques with monitoring at 280 nm (Fig 1), we assessed the isolation quality of MRJP1 oligomer, MRJP2 and MRJP3 by 2-DE with SYPRO Ruby staining (Fig 5). In the present experiments, separated MRJP data also agreed with previous reports [13, 16, 25], and other spots were not observed. These results indicate that separated MRJP1 oligomer, MRJP2 and MRJP3 were not contaminated by other proteins.

On 2-DE with CBB R-250 staining, the same 55-kDa protein spots ranging from pH 4.2 to 6.5 as shown in Fig 5A. Four of gel pieces were cut from the acidic side and subjected to MALDI TOF/TOF MS. After a search using ProteinPilot™ software (ver.4.5; AB sciex), these spots were identified as a MRJP1 of *Apis merifera*. In the same way, 52-kDa protein spots ranging from pH 6.2 to 7.9 (4 of gel pieces from the acidic side) as shown in Fig 5B and 60-72-kDa protein spots ranging from pH 7.4 to 8.2 (3 gel pieces from acidic side) as shown in Fig 5C were identified as MRJP2 and MRJP3 of *Apis merifera*, respectively (Table 1).

**Table 1:** Protein spots identified as MRJP1, MRJP2 and MRJP3.

A	Spot No.	Mw/pI	Accession No.	Coverage (%)*	Peptide (95%)*	Protein Name
	1	55kDa/4.2–6.5	O18330	26.2	7	MRJP1 *Apis merifera*
	2			41.7	13	
	3			39.1	11	
	4			39.1	13	
B	Spot No.	Mw/pI	Accession No.	Coverage (%)*	Peptide (95%)*	Protein Name
	1	52kDa/6.2–7.9	O77061	23.5	8	MRJP2 *Apis merifera*
	2			26.6	10	
	3			26.6	11	
	4			20.4	10	
C	Spot No.	Mw/pI	Accession No.	Coverage (%)*	Peptide (95%)*	Protein Name
	1	60-72kDa/7.4–8.2	Q17060	22.6	9	MRJP3 *Apis merifera*
	2			22.1	8	
	3			23.4	8	

No., number; Mw, moleculer weight; pI, isoelectric point.

*Data were acquired using MALDI-TOF/TOF (AB ciex). An MS/MS ion search was performed using ProteinPilot.

### MRJP1 is the main cell proliferation protein among soluble royal jelly proteins

**Fig 6:**
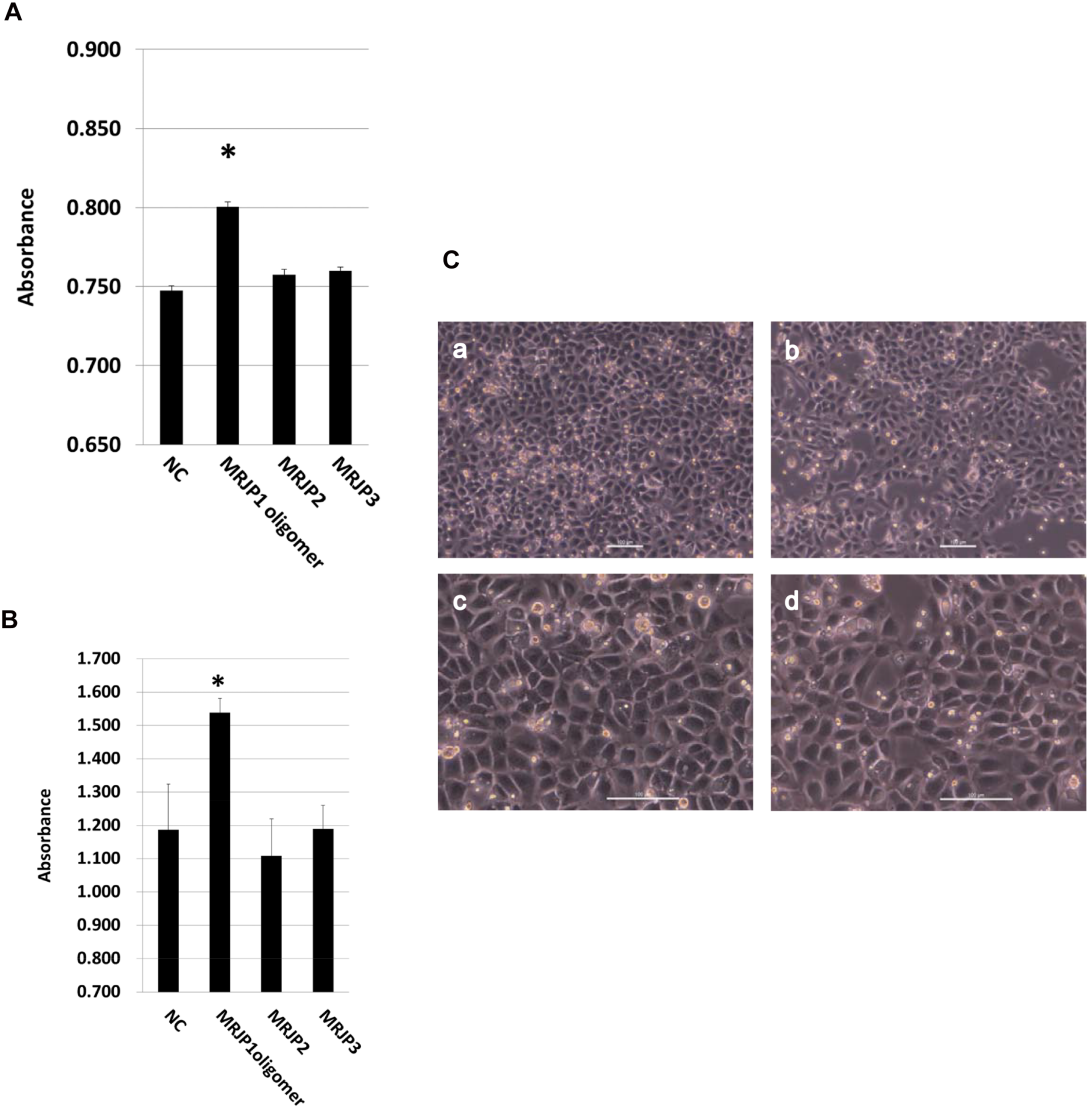
Cell proliferation activity of MRJP1 oligomer, MRJP2 and MRJP3. (A) Cell proliferation activity on Jurkat cells. Cells were cultured with DME/F-12 medium containing 0.5 mg/ml each protein for 24 hours. For negative control (NC), cells were cultured with DME/F-12 medium without any proteins. Cell proliferation was measured by Alamar Blue assay. Error bar indicate S.E.M. from three independent experiments (n = 3). Asterisk indicates p<0.01 vs NC. (B) Cell proliferation activity on IEC-6 cells. Cells were cultured in D-MEM medium containing 0.5 mg/ml each protein for 24 hours. For negative control (NC), cells were cultured with D-MEM medium without any proteins. Cell proliferation was measured by WST-8 assay. Error bar indicates S.E.M. from three independent experiments (n = 3). Asterisk indicates p<0.05 vs NC. (C) Phase-contrast images of IEC-6 cells. Cells were cultured in D-MEM medium containing 0.5 mg/ml MRJP1 (a, c) and without any protein (b, d) for 24 hours. Scale bar indicates 100μm. Magnification of figure a, b and figure c, d are 100-fold and 200-fold, respectively.

Soluble proteins are candidate factors for the mammiferous cell growth activity of RJ. However, proliferative activities of proteins other than MRJP1 oligomer have never been elucidated. Therefore, to characterize the function of “soluble RJ proteins”, we examined the proliferation activities of MRJP2 and MRJP3, as well as MRJP1 oligomer (Fig 6). Proliferation experiments based on Alamar blue assay using Jurkat cells (Fig 6A) and WST-8 assay using IEC-6 cell (Fig 6B). MRJP1 oligomer exerted significantly potent proliferation activity (p<0.01 and/or p<0.05, vs. negative control) in both assays, consistent with our previous data [8], whereas MRJP2 and MRJP3 did not promote cell growth in both Jurkat cell and IEC-6 cells. The phase-contrast images of IEC-6 cells culture analysis were shown in Fig 6C. These observations suggest that MRJP1 oligomer is the main protein responsible for the activity of proliferating mammiferous cells in soluble royal jelly proteins.

### Biochemical properties of MRJP1 oligomer against heat-treatment

**Fig 7:**
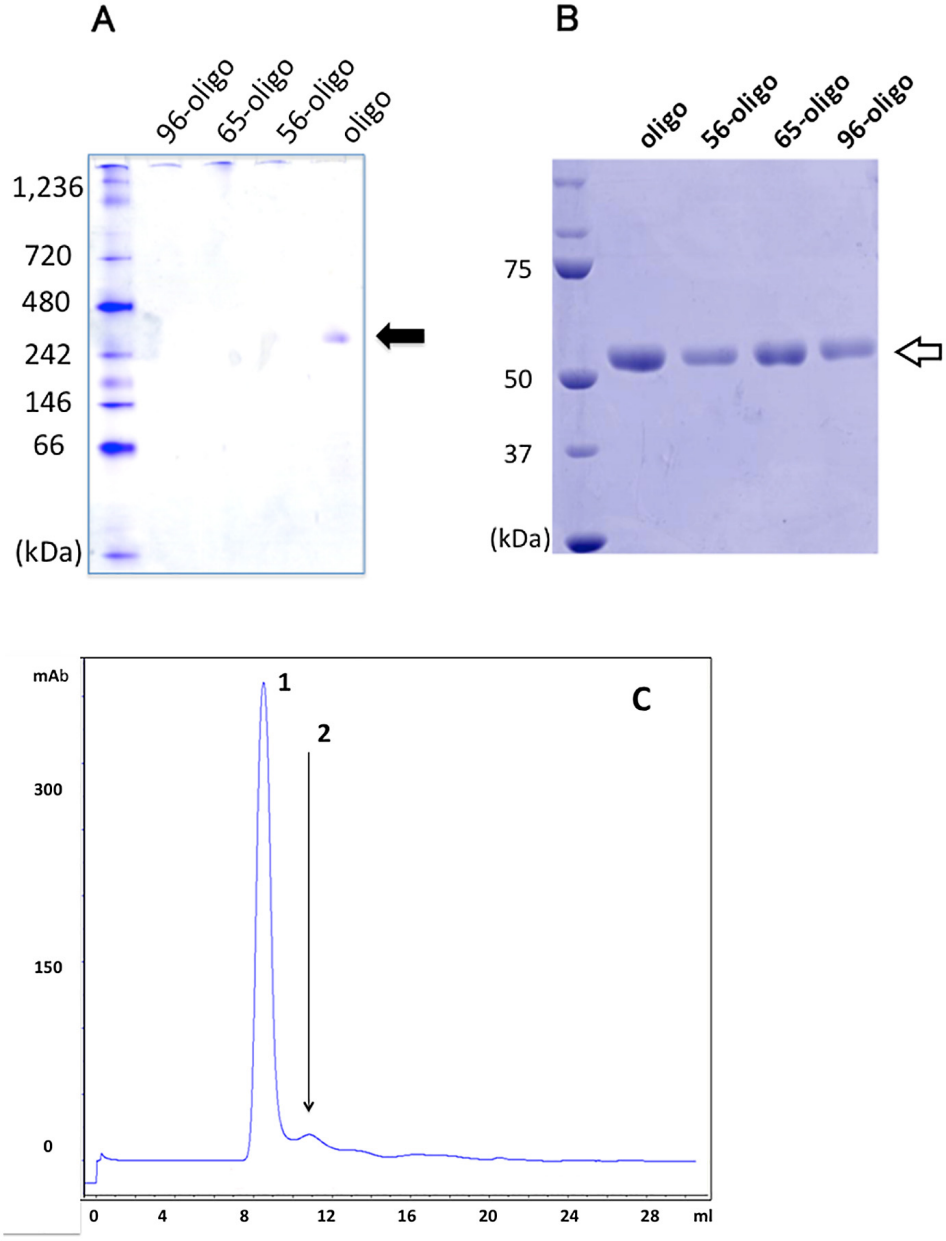
Biochemical feature of heat-treated MRJP1 oligomer. (A), Blue Native-PAGE analysis of heat-treated MRJP1 oligomer. A filled arrow indicates the MRJP1 oligomer, approximately 290kDa. (B), SDS-PAGE analysis of heat-treated MRJP1 oligomer. An open arrow indicates the 55kDa band of subunit from MRJP1 oligomer (MRJP1 monomer). Oligo indicates the MRJP1 oligomer without heat treatment. 56-oligo, 65-oligo and 96-oligo indicate the MRJP1 oligomer with 56°C, 65°C and 96°C treatment for 30min, respectively. Five micrograms of several analytes were applied to gels and visualized with CBB staining. (C), Superose 12 size-exclusion HPLC elution pattern of the 56-oligo sample. Peak 1 indicates heat aggregated MRJP1 oligomer at elution volume of 9 ml. Peak 2, indicated by the arrow, shows authentic elution peak of MRJP1 oligomer at elution volume of 11 ml. The column void volume was 7 ml.

In order to analyze the biochemical properties of MRJP1 oligomer against heat treatment, MRJP1 oligomers after heat treatment at various temperatures (56°C for 30 min: 56-oligo; 65°C for 30 min: 65-oligo; 96°C for 30 min: 96-oligo) were analyzed by Blue Native-PAGE and SDS-PAGE (Fig 7). On Blue Native-PAGE with CBB staining, band indicating MRJP1 oligomer (approximately 290 kDa) were detected in the lane of unheated MRJP1 oligomer (oligo). In contrast, the band of 56-oligo was not detected and/or it could be slightly observed. Those of 65-oligo and 96-oligo were not entirely detected. By heat-treated over 56°C, the intact MRJP1 oligomers were converted to heat-aggregated form. Then, the samples were remained on the origin of the Blue Native-PAGE gel and those samples were not able to enter into the gel (Fig 7A).

In contrast, SDS-PAGE analysis showed that 55-kDa bands, a subunit of the oligomeric complex known as MRJP1 monomer, were present in all analytes (Fig 7B). These samples (untreated MRJP1, 56-oligo, 65-oligo, and 96-oligo) were subjected to Superose 12 size-exclusion HPLC. As a result, a macromolecular peak, a heat-aggregated MRJP1 oligomer peak similar to heat aggregated human IgG [26, 27], was observed (refer to S1 Fig). Fig 7C shows the elution profile of the 56-oligo sample. The native MRJP1 oligomer was detected at the 11-ml position (approximately 280 kDa [24]; indicated by the arrow), but a macromolecular peak was also observed at the 9-ml position. In chromatographs for 65-oligo and 96-oligo, the macromolecular peak appeared at 8 ml (data not shown). These results indicate that little of the oligomeric form of MRJP1 was preserved at temperatures up to 56°C. However, the oligomeric forms were not detected in 65-oligo and 96-oligo samples. Therefore, on Blue Native-PAGE (Fig 7A), these samples of 65-oligo and 96-oligo were unable to enter into the gel. Therefore, the Blue Native-PAGE patterns (Fig 7A) agreed with the finding that heat-aggregated macromolecules of MRJP1 oligomer were present (Fig 7C). The same phenomenon was also confirmed on Superose 12 HPLC analysis, shown in S1 Fig, and Blue Native-PAGE of heat-aggregated human IgG.

However, the heat-aggregated MRJP1 oligomer was readily separated into 55-kDa monomeric bands by SDS-PAGE analysis (Fig 7B), probably as a result of conversion into the monomeric form by SDS buffer treatment.

### Proliferative activity of heat-treated MRJP1 molecules

**Fig 8:**
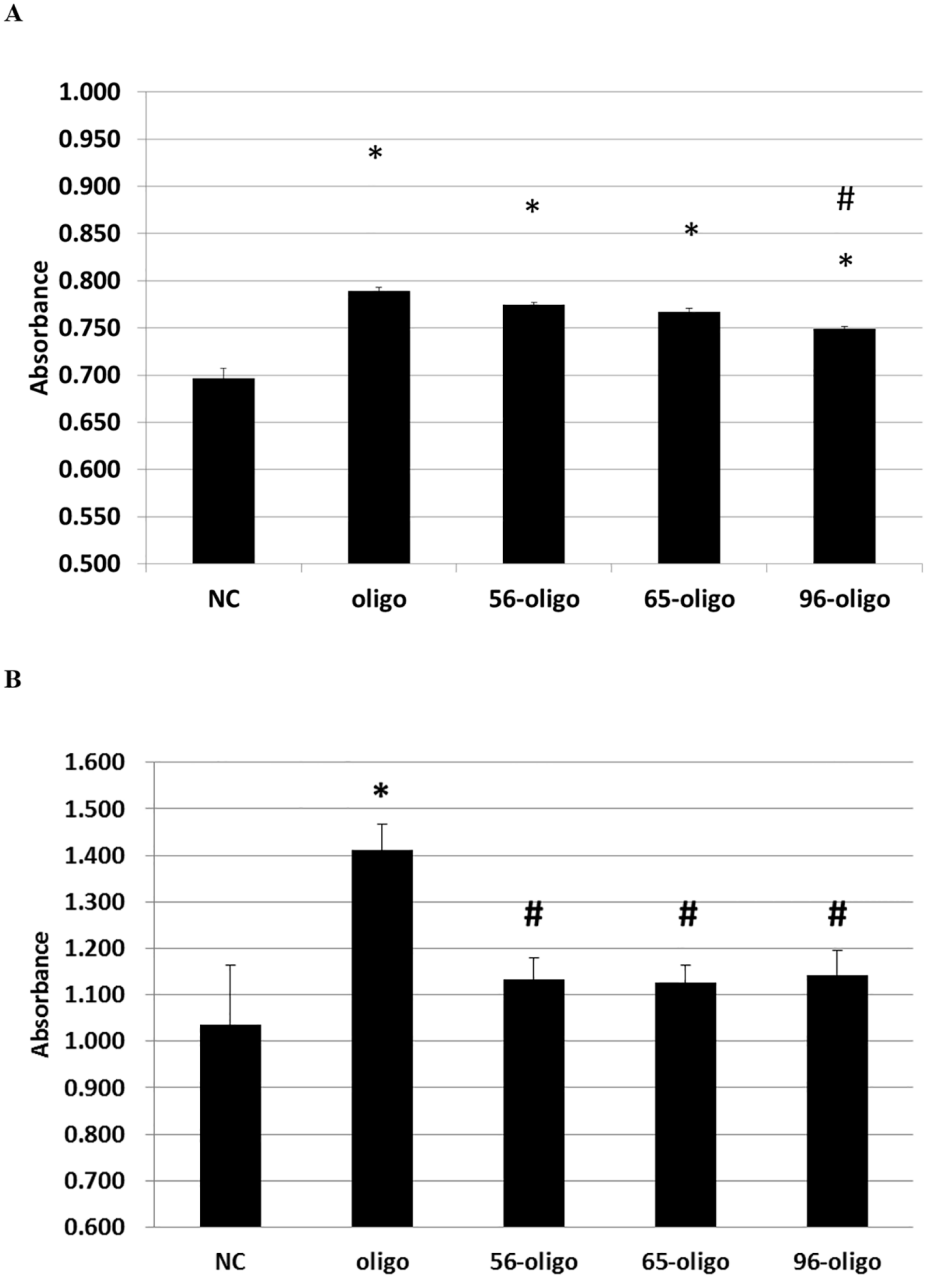
Jurkat Cell proliferation activity of heat-treated MRJP1 oligomers. (A) Jurkat cells were cultured with DME/F-12 medium containing 0.5 mg/ml heat treated MRJP1 oligomer for 24 hours. Oligo indicates the MRJP1 oligomer without heat treatment. 56-oligo, 65-oligo and 96-oligo indicate the MRJP1 oligomer with 56°C, 65°C and 96°C treatment for 30min, respectively. For negative control (NC), cells were cultured with DME/F-12 medium without any proteins. Cell proliferation was measured by Alamar Blue assay. Error bar indicate S.E.M. from three independent experiments (n = 3). Oligo indicates the MRJP1 oligomer without heat treatment. Asterisk indicates p<0.01 vs NC and pound sign indicates p < 0.01 vs oligo. (B) Cell proliferation activity on IEC-6 cells. Cells were cultured in D-MEM medium containing 0.5 mg/ml each protein for 24 hours. Oligo indicates the MRJP1 oligomer without heat treatment. 56-oligo, 65-oligo and 96-oligo indicate the MRJP1 oligomer with 56°C, 65°C and 96°C treatment for 30min, respectively. For negative control (NC), cells were cultured with D-MEM medium without any proteins. Cell proliferation was measured by WST-8 assay. Error bar indicate S.E.M. from three independent experiments (n = 3). Asterisk indicates p<0.01 vs NC and pound sign indicates p < 0.05 vs oligo.

Given the observation that an oligomeric form of MRJP1 was dissociated after heat treatment above 56°C, we subsequently investigated proliferation activities of MRJP1 molecules (Fig 8). On Alamar Blue assay using Jurkat cells, MRJP1 oligomer and heat-treated MRJP1 molecules (56-oligo, 65-oligo, and 96-oligo) induced clear increases in the number of cells. However, as compared to MRJP1 oligomer, the activity of 96-oligo was significantly lower (p < 0.01). These results suggest that the physiological activity of MRJP1 oligomer is resistant to heat treatment, but excessive heat shock, such as treatment at 96°C, is likely to attenuate its activity. In contrast, on WST-8 assay using IEC-6 cells, MRJP1 oligomer induced clear increases in the number of cells (p < 0.01) but those of heat-treated MRJP1 molecules were significantly lower vs MRJP1 oligomer (p <0.05). As a result, it was suggested that the cell proliferation effects of the heat treatment MRJP1 were indicated to be different by the origin of the cells.

## Discussion

In this study, to further characterize MRJP1, we investigated its biochemical and functional properties after heat treatment at various temperatures. The present results indicated that the bioactivity of MRJP1 oligomer was preserved after heat treatment, which suggests that MRJP1 oligomer is a heat-resistant protein. However, it was considered that its nature was different by the origin of the cells. In the future, we would like to also experiment from a different point of view.

As MRJP1 oligomer is the most abundant soluble royal jelly protein in royal jelly, we investigated the biochemical properties and functions of MRJP1 oligomer, thus demonstrating that MRJP1 oligomer stimulates human-derived cell proliferation [8]. However, the proliferative activities of other soluble royal jelly proteins (e.g., MRJP2 and MRJP3) have remained unclear. Therefore, prior to characterizing the heat resistance feature of MRJP1 oligomer, we first assessed the cell growth activities of MRJP2 and MRJP3, as well as MRJP1 oligomer. Cell proliferation assay showed that MRJP1 oligomer, but not MRJP2 and MRJP3, significantly increased cell numbers (Fig 6), suggesting that MRJP1 oligomer is the predominant proliferation factor for mammiferous cells among the abundant soluble royal jelly proteins.

MRJP1 oligomer is considered to be responsible for the cell growth activity of royal jelly; thus, we subsequently performed heat treatment experiments focusing on MRJP1 oligomer. We previously reported that MRJP1 oligomer maintained its oligomeric conformation after heat treatment at 56°C, but not at 96°C [8]. However, it has not been investigated whether these heat-treated MRJP1 oligomers, defined as “MRJP1 molecules” in this report, show altered physiological activity. Electrophoretic and size-exclusion HPLC analysis showed that MRJP1 oligomer remains little amount of its oligomeric form until 56°C and the heat-aggregated form was observed at the same temperature. In contrast, the samples of 56-oligo, 65-oligo and 96-oligo were readily separated by SDS-PAGE. Bilikova and Simuth [28] also reported that those heat characteristics of apalbunin 1 were confirmed by the method of immunoblotting. They demonstrated that the immunoblotting patterns after heating at 30, 60 and 80°C were not changed in comparison with the unheated control sample. Our SDS-PAGE data for 56-oligo, 65-oligo and 96-oligo agreed with the immunoblotting data. On the other hand, heat-aggregated proteins are known among human IgG [26], bovine serum albumin [29], and ovalbumin [30]. In these heat-aggregated proteins, the basic activities are maintained and new activities are observed.

With regard to proliferation activity of MRJP1 molecules, the 56-oligo, 65-oligo and 96-oligo forms all showed increases in cell number (Fig 8). However, when compared to unheated MRJP1 oligomer, the activity of 96-oligo was attenuated. We also performed the cell proliferation assay on 56-oligo and 96-oligo for 48 hours (data not shown).

In this assay, 56-oligo provoked further cell proliferation activity from 24 hours to 48 hours, but this further proliferation was not observed with 96-oligo. Previously, Watanabe et al. found that a protein from royal jelly, termed DIII protein, showed heat-resistant cell growth activity [12], although excess high temperatures, such as 100°C, caused decreased bioactivity of DIII protein. It is unknown which protein is referred to as DIII protein in the NCBI gene database. However, we believe that the data on DIII protein is consistent with the present results on the heat-resistant properties of MRJP1 oligomer, and thus, it is possible that DIII protein is MRJP1 oligomer. On the other hand, the functional mechanisms of MRJP1 oligomer or monomer for human derived cells remain elusive. However, previous studies on recombinant MRJP1 monomer synthesized from *E*. *coli*, yeast and insect cells have suggested that the sugar chain on MRJP1 is an essential factor for exerting cell proliferation activity [31, 32]. We therefore consider that excess temperature can alter the sugar chains of MRJP1 molecules, which results in attenuation of proliferation activity.

With regard to the application of the present data to industrial uses of royal jelly, the heat-resistance properties of MRJP1 oligomer are beneficial for food sanitation. The functional resistance of MRJP1 oligomer against moderate heat treatment (up to 65°C) indicates that MRJP1 oligomer is suitable for application to low-temperature pasteurization. At present, various liquid food products (e.g., wine, milk and beer) are pasteurized. Therefore, application of royal jelly to low-temperature pasteurization would greatly contribute to the supply of safe royal jelly and the development of health food products based on royal jelly. In contrast, Shen et al. [33] recently reported that the well-known antibacterial substance royalisin [34] was weakened by heat treatment ranging from 55°C to 85°C for 15 min in studies with recombinant proteins.

The limitations of this study include the fact that we focused on only three soluble royal jelly proteins (MRJP1 oligomer, MRJP2 and MRJP3)) in order to characterize the cell growth activity of RJ. The MRJP family contains other members, MRJP4-9, and soluble royal jelly proteins also contain numerous other proteins at low levels. As these unknown RJ proteins may also stimulate cell growth, further analyses based on deep-identification proteomics are required in order to more accurately assess the physiological functions of royal jelly. Furthermore, in this study, we used the human-derived cell line (Jurkat cells) and rat-derived cell line (IEC-6 cells) for the cell proliferation assay. To more precisely evaluate the function of RJ in humans, use of a primary cell line is necessary. Finally, for the physiological function of MRJP1 oligomer, we only analyzed proliferation activity after heat treatment. It is reported that MRJPs also have other functions, such as immune response amelioration [18, 35–37] and antimicrobial activity [38, 39]. It is necessary to carefully examine whether these functions are also altered upon heat treatment.

In conclusion, MRJP1 oligomer, the main proliferation factor in royal jelly, is able to biochemically and functionally resist moderate heat treatment. Although excess heat shock, such as 96°C, causes attenuation of cell growth activity by MRJP1 oligomer, the heat-resistance properties of MRJP1 oligomer should allow royal jelly to be subjected to pasteurization, which will contribute to the advancement of food sanitation of royal jelly products.

## Supporting Information

S1 Fig Superose 12 size-exclusion HPLC elution pattern of heat aggregated human IgG. Purified IgG solution (10 mg/ml) was subjected to heat-treatment (65°C, 30 min) and 0.2 ml of the sample was applied to the column. Peak 1 and 2 indicate macromolecular form of heat aggregated IgG. Peak 3 indicates authentic elution peak of human IgG (150 kDa). The column void volume was 7 ml. Purified IgG (Sigma Aldrich) solution (10 mg/ml) was subjected to heat-treatment at 65°C for 30 min. Then, the sample was analyzed by Superose 12 size-exclusion HPLC and Blue Narive-PAGE (4–16% bis-Tris gel, Invitrogen). The aggregated IgG prepared with size-exclusion chromatography has been used to as a standard material in the analysis of circulating immune complexes[26, 27, 40]. In Blue Narive-PAGE, the fraction of heat aggregated IgG was not entered into the gel as heat-treated MRJP1 oligomer over 56°C.
(TIFF)
