# Supplementary figures and images for: Heat Resistant Characteristics of Major Royal Jelly Protein 1 (MRJP1) Oligomer
Source: PLoS One. 2015 May 28;10(5):e0119169. doi: 10.1371/journal.pone.0119169 (PMC4447272; doi:10.1371/journal.pone.0119169)

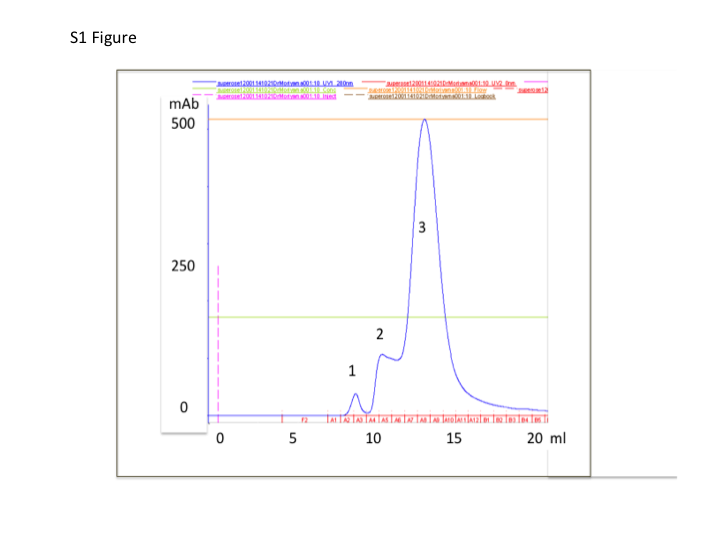

Supplement: S1 Fig — Purified IgG solution (10 mg/ml) was subjected to heat-treatment (65°C, 30 min) and 0.2 ml of the sample was applied to the column. Peak 1 and 2 indicate macromolecular form of heat aggregated IgG. Peak 3 indicates authentic elution peak of human IgG (150 kDa). The column void volume was 7 ml. Purified IgG (Sigma Aldrich) solution (10 mg/ml) was subjected to heat-treatment at 65°C for 30 min. Then, the sample was analyzed by Superose 12 size-exclusion HPLC and Blue Narive-PAGE (4–16% bis-Tris gel, Invitrogen). The aggregated IgG prepared with size-exclusion chromatography has been used to as a standard material in the analysis of circulating immune complexes[26, 27, 40]. In Blue Narive-PAGE, the fraction of heat aggregated IgG was not entered into the gel as heat-treated MRJP1 oligomer over 56°C. (TIFF) [file pone.0119169.s001.tiff]
